# The Quebec Dental Anomalies Registry: Identifying genes for rare disorders

**DOI:** 10.1093/pnasnexus/pgad196

**Published:** 2023-06-14

**Authors:** Madeleine S Wredenhagen, Andee Goldstein, Hélène Mathieu, Valancy Miranda, Burcin Morali, Jacinthe Santerre, Catalina Maftei, Marie-Ange Delrue, Matthieu Schmittbuhl, Duy Dat Vu, Florina Moldovan, Philippe M Campeau

**Affiliations:** CHU Sainte-Justine Research Center, 3175 Chemin de la Côte-Sainte-Catherine, Montreal, QC, Canada, H3T1C5 and University of Ottawa, 75 Laurier Ave E, Ottawa, ON, Canada K1N 6N5; CHU Sainte-Justine Research Center, 3175 Chemin de la Côte-Sainte-Catherine, Montreal, QC, Canada, H3T1C5 and Université de Montréal, 2900 Edouard Montpetit Boulevard, Montreal, QC, Canada, H3T1C5; CHU Sainte-Justine Research Center, 3175 Chemin de la Côte-Sainte-Catherine, Montreal, QC, Canada, H3T1C5 and Université de Montréal, 2900 Edouard Montpetit Boulevard, Montreal, QC, Canada, H3T1C5; Department of Pediatrics, CHU Sainte-Justine Research Center, 3175 Chemin de la Côte-Sainte-Catherine, Montreal, QC, Canada, H3T1C5; Department of Pediatrics, CHU Sainte-Justine, 3175 Chemin de la Côte-Sainte-Catherine, Montreal, QC, Canada, H3T1C5; Department of Pediatrics, CHU Sainte-Justine, 3175 Chemin de la Côte-Sainte-Catherine, Montreal, QC, Canada, H3T1C5; CHU Sainte-Justine, Genetic Service, 3175 Chemin de la Côte-Sainte-Catherine, Montreal, QC, Canada, H3T1C5; Department of Pediatrics, CHU Sainte-Justine, 3175 Chemin de la Côte-Sainte-Catherine, Montreal, QC, Canada, H3T1C5; Faculty of Dentistry, Department of Stomatology, Université of Montréal, 2900 Edouard Montpetit Boulevard, Montreal, QC, Canada H3T 1J4; Faculty of Dentistry, Université of Montréal, 2900 Edouard Montpetit Boulevard, Montreal, QC, Canada H3T 1J4; CHU Sainte-Justine Research Center and Faculty of Dentistry, Department of Stomatology, Université de Montréal, 2900 Edouard Montpetit Boulevard, Montreal, QC, Canada, H3T1C5; Department of Pediatrics, CHU Sainte-Justine Research Center, 3175 Chemin de la Côte-Sainte-Catherine, and Université de Montréal, 2900 Edouard Montpetit Boulevard, Montreal, QC, Canada, H3T1C5

**Keywords:** genetics, craniofacial anomalies, pediatric dentistry, oligodontia

## Abstract

There are more than 900 genetic syndromes associated with oral manifestations. These syndromes can have serious health implications, and left undiagnosed, can hamper treatment and prognosis later in life. About 6.67% of the population will develop a rare disease during their lifetime, some of which are difficult to diagnose. The establishment of a data and tissue bank of rare diseases with oral manifestations in Quebec will help medical professionals identify the genes involved, will improve knowledge on the rare genetic diseases, and will also lead to improved patient management. It will also allow samples and information sharing with other clinicians and investigators. As an example of a condition requiring additional research, dental ankylosis is a condition in which the tooth's cementum fuses to the surrounding alveolar bone. This can be secondary to traumatic injury but is often idiopathic, and the genes involved in the idiopathic cases, if any, are poorly known. To date, patients with both identified and unidentified genetic etiology for their dental anomalies were recruited through dental and genetics clinics for the study. They underwent sequencing of selected genes or exome sequencing depending on the manifestation. We recruited 37 patients and we identified pathogenic or likely pathogenic variants in *WNT10A*, *EDAR*, *AMBN*, *PLOD1*, *TSPEAR*, *PRKAR1A*, *FAM83H, PRKACB, DLX3, DSPP, BMP2, TGDS*. Our project led to the establishment of the Quebec Dental Anomalies Registry, which will help researchers, medical and dental practitioners alike understand the genetics of dental anomalies and facilitate research collaborations into improved standards of care for patients with rare dental anomalies and any accompanying genetic diseases.

**Significance Statement**

The results of this study describe both the clinical presentation of patients and the genes found to be associated with their conditions and will help practitioners recognize the possibility of an underlying genetic condition by focusing on the dental anomalies, and, in turn, provide patients with earlier diagnoses and better treatments.

## Introduction

There are over 900 syndromes associated with oral manifestations. The full list is present on websites such as OMIM, and a detailed description of selected syndromes can be found on our project's website (http://rd-dental.org/). About 1 in 15 individuals will develop a rare disease (6.67% of the population), and of those disorders, 80% will be transmitted genetically (1). Dental manifestations are often a result of a syndrome affecting multiple systems, which can be detected notably by dentists. The objective of the data and tissue bank is to collect information and samples on consented patients as to their clinical characteristics and their genetic test results and conduct additional genetic tests if necessary. This will expand and improve our knowledge of these anomalies and associated syndromes. No such data or tissue bank currently exists in Quebec. The benefits of the bank are to facilitate open collaboration between medical professionals, to share information and samples, resolve existing cases, discover new genes for dental anomalies, as well as to help practitioners in their fields by recognizing the possibility of an underlying genetic condition simply by means of dental information.

Dental anomalies can sometimes be influenced by environmental factors, but the susceptibility of developing the condition increases with the presence of variants in certain key genes. Dental erosion, for example, is not a condition with a single cause—it can be influenced by the composition of the saliva, a person's eating and drinking habits, as well as the composition of the tooth's enamel. Variants in the genes responsible for enamel formation would increase the chances of being affected (2). *AMBN* is associated with the tooth's enamel (3) and its variants are also associated with amelogenesis imperfecta (AI) (Type 1F) which is a group of genetic conditions that affect the enamel of more or less all the teeth equally and can be associated with other changes elsewhere in the body (4).

Other anomalies have an uncertain etiology and there is a lot of critical information about such conditions that could be understood from dissecting the genetics involved. Ankylosis, for example, is a condition where the tooth's cementum fuses to the surrounding alveolar bone, rendering the tooth immobile affecting the eruption pattern on the teeth (5).

Moreover, the same gene might be responsible for multiple conditions, such as a *WNT10A* variant, which can result in oligodontia, ectodermal dysplasia, or more severe syndromes (6). The same condition might also be caused by two different genes, such as ectodermal dysplasia [a group of genetic disorders affecting the tissues derived from the ectoderm such as teeth, hair, nails, skin, and eccrine glands (7)] also being caused by *EDAR* variants.

Similarly, the severity of the affected individual's phenotype can vary widely depending on the variants of a same gene, such as *TSPEAR* (important for tooth and nail development), which can be responsible for ranging severities of associated syndromes (8).

In this study, patients with both identified and unidentified genetic etiologies were recruited through dental and genetics clinics for the study. We recruited 37 patients in whom we identified pathogenic or likely pathogenic variants in 12 genes: *WNT10A*, *EDAR*, *AMBN*, *PLOD1*, *TSPEAR*, *PRKAR1A*, *FAM83H, PRKACB, DLX3, DSPP, BMP2, TGDS*.

By understanding the underlying genetics of dental anomalies and identifying new genes or genetic variants in patients with dental anomalies, we can help patients with earlier diagnoses and better treatments.

## Results

The 37 patients (from 25 families) enrolled in this study represented various category of dental anomalies such as oligodontia, dentinal dysplasia, ectodermal dysplasia, and so on. The clinical description of each patient and their families (if relevant) is presented in Online Supplementary Table S1 (TS1). No patient withdrew from our study.

### Overview of molecular studies

Our registry is still enrolling, and we are still conducting molecular studies. So far, of the 25 families, pathogenic or likely pathogenic variants were identified in 19 families. Seventeen families had panel testing on a clinical basis and in those, pathogenic or likely pathogenic variants were identified in 15, while for 2, only variants of unknown significance (VUSs) were identified. Additionally, six families directly had exome sequencing on a research basis; likely pathogenic variants were identified in four (but familial segregation needs to be done in two), whereas in two other families, there were no candidate genes on exome sequencing. Finally, for Family 11, only *DSPP* was sequenced and is negative, and for Family 18, molecular tests have not yet been performed. Therefore, additional tests such as trio exome or genome sequencing may still be indicated in at least six families.

Below is a breakdown of our cohort according to the main dental anomalies.

*Oligodontia* (a rare genetic disorder which represents the congenital absence of more than six teeth in primary, permanent, or both dentitions) was found in 14 of 37 patients.

Three patients (siblings) presented with oligodontia and a family history of oligodontia. They were found to be compound heterozygous for pathogenic and likely pathogenic (respectively) *WNT10A* variants (NM_025216.3: c.803C > G; p.Ser268Ter and c.682T > A; p.Phe228Ile). These variants are compatible with a recessive Odonto-onycho-dermal dysplasia diagnosis (Family 1).

Another patient presenting with isolated oligodontia with congenital absence of seven teeth and thin hair was found to have a heterozygous variant in the *WNT10A* gene (NM_025216.3: c.511C > T; p.Arg171Cys). We consider this variant as likely pathogenic acting dominantly with reduced penetrance, given that the patient's mother is a carrier for a *WNT10A* variant and there is a family history of oligodontia (Family 2).

A heterozygous dominantly acting likely pathogenic de novo *WNT10A* (NM_025216.3: c.283G > A; P.Glu95Lys) variant was found in a patient with isolated oligodontia, congenital absence of eight teeth, primary teeth retention, dental malocclusion (as well as shape anomalies), and a moderated lack of alveolar growth (Family 3).

Dizygotic twins, one presenting with oligodontia with absence of 11 to 15 permanent teeth, global delayed development, asthma, pneumonias, hypospadias, dysplastic nails, excessive sweating of the feet, pectus excavatum, and spatulated fingers. The patient was diagnosed with Oligodontia Syndrome, and has two variants in the *WNT10A* gene (c.682T > A and NM_025216.3: c.1086C > A; p.Cys362Ter), determined to be likely pathogenic. The other twin, who presented with oligodontia with absence of 6 to 8 permanent teeth and hyperlaxity in the small articulations, also had the same two variants (Family 4).

Two variants in the *WNT10A* gene have been identified in a patient with Odonto-onycho-dermal Syndrome. The patient presented multiple dental anomalies, oligodontia, hyperlaxity, clinodactyly of the fifth finger, excessive sweating of the hands and feet, dry and brittle hair, flat fragile and striated nails, and deformed pointy primary teeth. Exome sequencing identified two variants in *WNT10A* (NM_025216.3: c.321C > A; p.Cys107Ter) and c.803C > G (p.Ser268Ter), and the variants were determined to be in trans (Family 8).

A heterozygous likely pathogenic *WNT10A* c.682T > A variant has been found in a patient presenting with oligodontia with agenesis of 10 adult, consistent with a diagnosis of multiple tooth agenesis Type 4. The patient also presented with Attention deficit hyperactivity disorder (ADHD), eczema, anxiety, and bilateral clinodactyly of the third finger (Family 13).

Exome sequencing identified a pathogenic heterozygous variant in the *WNT10A* gene (NM_025216.3: c.1237G > A; p.Val413Ile) in a patient presenting with multiple dental agenesis with congenital absence of six teeth (four premolars on the upper jaw and two on the lower jaw), retention of primary teeth, fragile nails, fructose intolerance, dermographism, and hallux valgus (Family 14).

A patient presented with oligodontia (missing five teeth and wisdom teeth), retrognathia, a narrow and high-arched palate, slight gingival hypertrophy, predominant thenar hypertrophy of the left side, slight muscular hypotrophy of the left forearm, small fingers with some having a pronounced hyperlaxity. He also had limitations in the flexibility of the wrists, short and stocky feet with a metatarsus varus positional tendency, hyperhidrosis of the hands and feet with a slight plantar hyperkeratosis, distal arthrogryposis of the upper limbs and glasses at a young age to correct astigmatism. A heterozygous VUS *WNT10A* (NM_025216.3: c.715C > T; p.His239Tyr) variant of maternal origin was found in one patient (Family 25).

A pathogenic heterozygous *WNT10A* c.803C > G (p. Ser268Ter) variant was found in a patient presenting with oligodontia of 2 primary teeth and 11 permanent teeth of nonsyndromic appearance (Family 15).

A patient presenting with an ankylosed tooth (#85) as well as the congenital absence of tooth #45 was found to be heterozygous for a likely pathogenic *PRKACB* (NM_182948.4): c.1000A > C variant (p.Asn334His). *PRKACB* is associated with a syndromic oligodontia. Familial segregation and thorough phenotyping are pending (Family 22).

We recruited a patient with oligodontia with congenital absence of nine teeth, but no candidate variant has been identified in this patient yet (Family 16).

*Dentinal dysplasia*, a rare disturbance of dentin formation characterized by normal enamel but atypical dentin formation with abnormal pupal morphology, was found in 1 of 37 enrolled patients. This patient presented dentinal dysplasia, incorrect orientation of the permanent dentition (due to premature eruptions), supernumerary teeth at the level of the incisors with “ghost” roots, thrombosis of the Galen vein, and delayed fine motor skills without signs of intellectual disability. The patient had no primary dentition defects other than a few cavities, and there was no family history of dental anomalies. These symptoms are consistent with Type 1 dentinal dysplasia. Sequencing of the *DSPP* gene was negative; however, the test did not include sequencing of the large repeat element found in exon 5 (Family 11).

*Ectodermal dysplasia*, a rare group of inherited disorders characterized by anomalies of ectodermal derived structures, was found in 5 of 37 patients.

The pair of siblings presented with ectodermal dysplasia had a family history of the disease. They were found to be heterozygous for a likely pathogenic *EDAR* variant (NM_022336.4: c.1234C > T; p.Leu412Phe), which has been determined to be the most probable cause of the familial ectodermal dysplasia (Family 6).

A patient with ectodermal dysplasia (conical spaced teeth, delayed eruption of certain teeth, but with all teeth present), thin hair, and fragile dysplastic nails on both hands and feet was found to have a pathogenic homozygous variant on the *WNT10A* gene (c.321C > A). The affected mother also has the variant (Family 7).

**Fig. 1.**
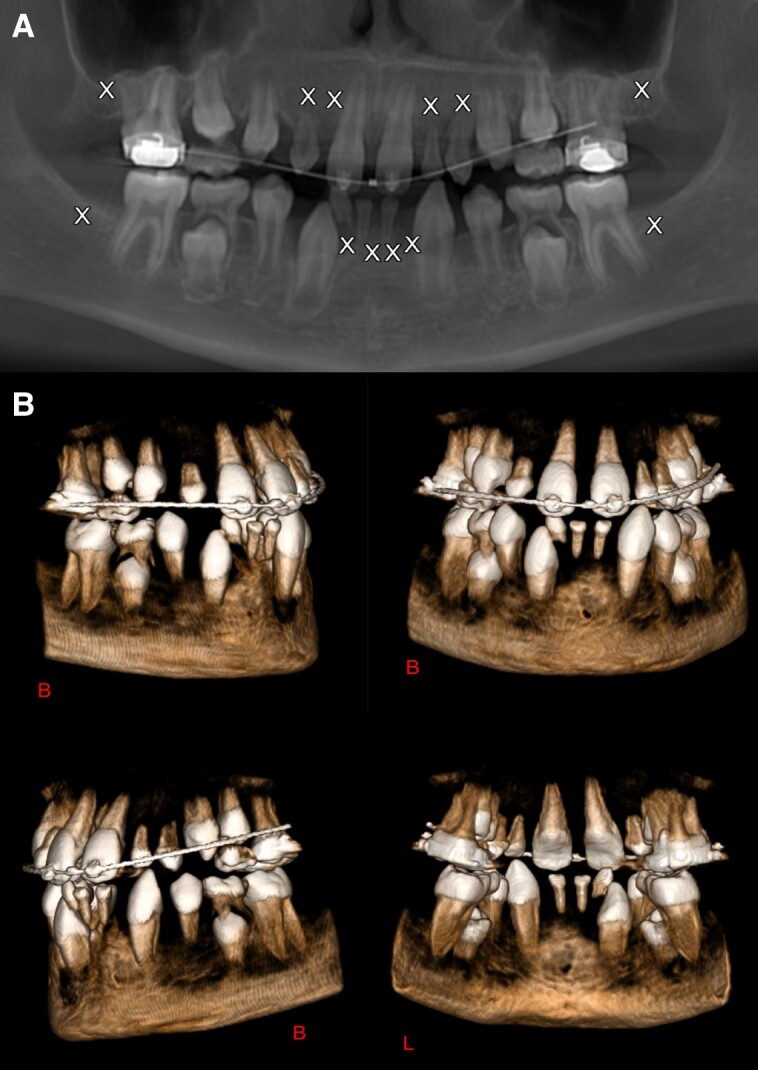
Cone beam imaging of a homozygous *TSPEAR* variant c.974delC; p.Ser325Ter in Individual 1, Family 10, TS1. Panoramic view A) and volume rendering B) of the dental arches. Severe oligondontia with 12 missing teeth; cone-like shapes of the lower canines (#33 and #43) and upper first premolars (#14 and #24).

A homozygous pathogenic variant in *TSPEAR* (NM_144991.3: c. 974delC) was found in a patient with severe oligodontia with 12 missing adult teeth, as well as cone-like shapes of the canines and premolars, root dysmorphism with distolingual roots on each molar (#16 and #26), and dental rhizalysis of teeth #65 and #75. However, there was no significant evidence for dental pulp anomalies especially around the molars and no evidence for osseous anomalies at the maxillary level. The patient also presents with ectodermal dysplasia, microdontia, and a supernumerary lower tooth. The patient had alacrima, late hair growth (age 5), and thin nails. The patient's phenotype is consistent with ectodermal dysplasia (Family 10) (Fig. 1).

A heterozygous microduplication of uncertain significance of at least 6.6 kb including the entire *KRT85* gene was found in a patient with microdontia, oligodontia (only eight adult teeth present), wiry hair, but with no evidence of affected skin. There is a positive family history, with the patient's sibling experiencing hair loss and skin problems, and the patient's father missing four adult teeth with early baldness (beginning at 20 years of age). Ectodermal dysplasia seemed the most probable cause and an ectodermal dysplasia panel identified the microduplication. Given that the ectodermal dysplasia associated with *KRT85* is a recessive condition, that a variant was identified on only one allele and that it is not clearly pathogenic, this patient's condition remains unsolved (Family 5).

**Fig. 2.**
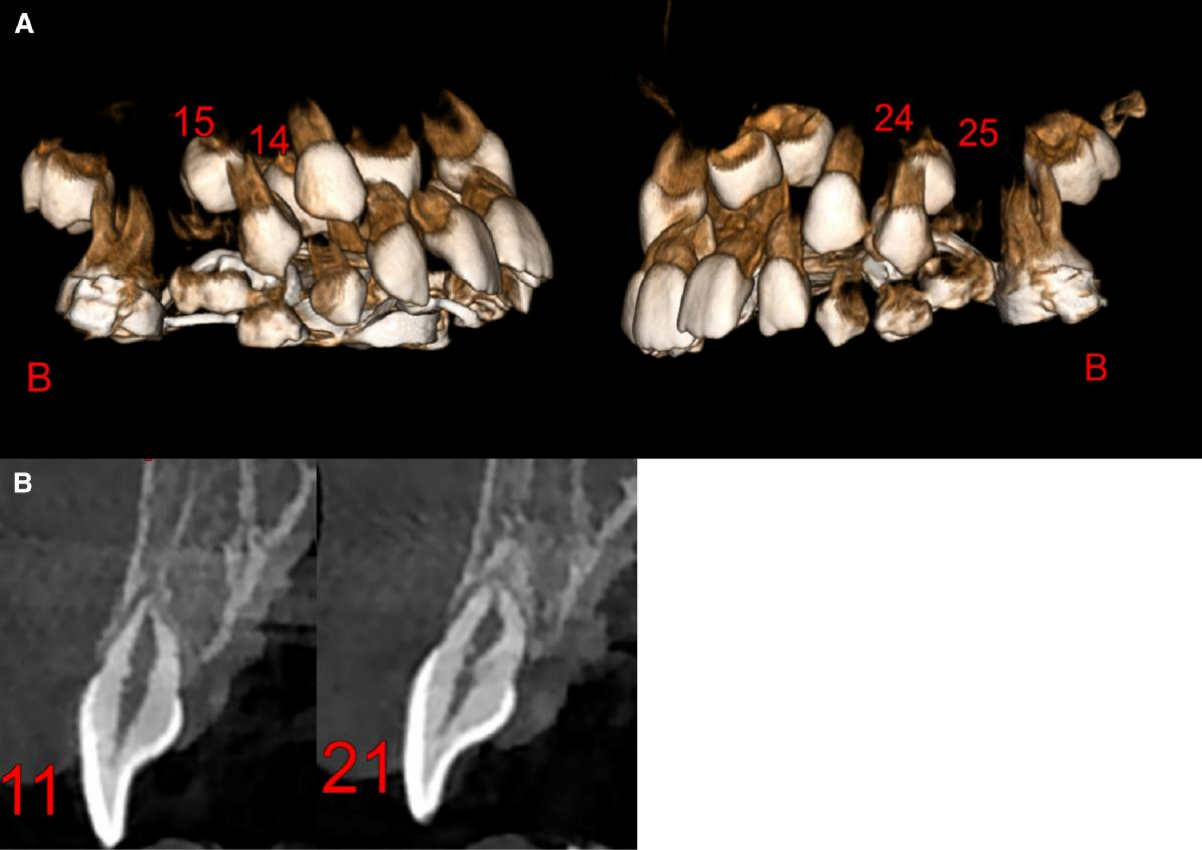
Cone beam imaging of a heterozygous *PRKAR1A* variant c.379_380delinsTT; p.Ala127Phe in Individual 1, Family 12, TS1. Volume rendering of the upper dental arch A) and buccolingual slices passing, respectively, through the upper central incisors (#11–21). Transposition tendency of germs #15 and #25; B) root abnormalities with short root portions of teeth #11–21.

*Dental ankylosis* was found in two patients. One of the patients presenting with ankylosis also has acrodysostosis and was found to have a likely pathogenic heterozygous *PRKAR1A* variant (NM_212472.2: c.379_380delinsTT; p.Ala127Phe). All the permanent teeth germs were present and there were no delayed eruptions. The superior incisor showed evidence of root dysmorphisms, notably short root portions of teeth #11–21. There was transposition tendency of germs #15–25 in relation to germs #14–24 (Family 12) (Fig. 2). Another patient presented with an ankylosed tooth (#85) and a congenital missing #45 tooth. No candidate variant has been identified in this patient yet (Family 17).

*Amelogenesis imperfecta* (AI): A patient with a seemingly isolated AI was recruited for the study and found to have a heterozygous VUS of *FAM83H* (NM_198488.5: c.1436G > A; p.Gly479Asp), a gene involved in autosomal dominant AI as well as a heterozygous 31.3 kb deletion in the *ITGB6* gene, a gene involved in autosomal recessive AI. Screening in family members is pending (Family 19). A third patient with AI of all primary teeth was found to be heterozygous for a *DLX3* (NM_005220.3: c.376C > T; p.Pro126Ser) variant. The findings may be consistent with Trichodentoosseous Syndrome. Familial segregation and thorough phenotyping are pending (Family 20).

Finally, *other anomalies* were found. A patient presenting with severe dental erosion and with a history of multiple fractures at a young age was found to be heterozygous for a pathogenic variant of the *AMBN* gene (NM_016519.6: c.209C > G; p.Ser70Ter). The patient is also heterozygous for a *PLOD1* missense splice region VUS (NM_000302.4: c.1097C > T; p.Ala366Val). Segregation remains to be done for both variants (Family 9).

A patient presenting with a narrow jaw, late apparition of adult teeth and a tooth alignment problem also presented with scoliosis, hallux valgus, a cranial parameter on the larger limit of the normal, a narrow palate, a bifid uvula, inverted nipples, and short and wide hands with possible brachydactyly. A pathogenic heterozygous *BMP2* (NM_001200.4: c.142G > T; p.Glu48Ter) variant was found and determined to be responsible for “Short stature, facial dysmorphism, and skeletal anomalies with or without cardiac anomalies 1” syndrome in this patient (Family 23).

A patient with malocclusion, dental extraction, misalignment of inferior teeth, arterial hypertension, bilateral clinodactyly also presented with a severe Pierre-Marie-Robin sequence with a cleft palate, difficulties with feeding which required gavage at a young age, vesicoureteral reflux at a young age, staturo-ponderal growth delay, slight developmental delay, and a large cardiac ventricular septal defect with a slight pulmonary stenosis. Two (compound heterozygous) likely pathogenic *TGDS* variants were identified: NM_014305.4: c.298G > T (p.Ala100Ser) and c.269A > G (p.Glu90Gly) (Family 24). This is consistent with Catel-Manzke Syndrome.

A patient presenting with dentinogenesis imperfecta was found to be heterozygous for a likely pathogenic *DSPP* frameshift variant (NM_014208.3: c.1439_1454del; p.Glu480AlafsTer829). Genetic analysis of the family members remains to be done (Family 21).

Finally, we recruited a patient presenting with a peg-shaped tooth (#22) and three congenital missing teeth. Genetic analyses have not yet been performed (Family 18).

## Discussion

Here, we describe the variants identified in various genes (*WNT10A*, *EDAR*, *AMBN*, *PLOD1*, *TSPEAR*, *PRKAR1A*, *FAM83H, PRKACB, DLX3, DSPP, BMP2, TGDS*) found in the 37 recruited patients presenting with diverse dental anomalies. Novel variants (or at least variants not reported in ClinVar) were found in multiple genes presented in our patients. A summary of the variants found in this study is presented in Online Supplementary Table S3.

The gene *WNT10A* plays an important role in early tooth development alongside several other genes such as *MSX1, PAX9, AXIN2, EDA*, and *EDARADD* (6, 9–13). van den Boogaard et al. (6) demonstrated that variants in this gene are present in 56% of patients with nonsyndromic tooth agenesis and are also associated with maxillary and mandibular molar agenesis and maxillary lateral incisor agenesis. Therefore, variants in *WNT10A* are a frequent cause of isolated hypodontia. The yield of molecular testing of isolated hypodontia increased from 15 to 71% by including *WNT10A* in the DNA diagnostics (6). We recruited 16 patients with various *WNT10A* variants, encompassing multiple dental anomalies. One of the two nonsense variants (c.321C > A; p.Cys107Ter) has been found in other patients (9). The *WNT10A* alleles producing only truncated proteins explain the severity of dental agenesis that we observed in the patient of Family 8. A heterozygous VUS *WNT10A* (NM_025216.3: c.715C > T; p.His239Tyr) variant of maternal origin was found in one patient (Familly 25). The mother however was asymptomatic other than missing wisdom teeth, thus there could be intrafamilial variability. It was determined that the patient's hyperhidrosis and oligodontia could potentially be caused by this variant of unknown significance, but no variant was found to explain the joint anomalies. Notably, patients with *WNT10A* variants most often presented with oligodontia [missing more than six teeth of primary, permanent, or both dentitions (6, 9)].

Dentinogenesis imperfecta is categorized into three types varying in degrees of severity of hereditary dentin defects. Type II describes a discolored dentition and bulbous crown shape, among others, similarly Type III describes a more severe form of Type II dentinogenesis imperfecta (13). The *DSPP* gene encodes for a noncollagenous protein in the dentin matrix cleaved into three major proteins. *DSPP* variants have been shown to cause Type II and Type III dentinogenesis imperfecta and dentin dysplasia II, with other candidate genes possibly causing those conditions as well (13). This suggests that these three diseases are on a same continuum of varying degrees (14). A patient presenting with dentinogenesis imperfecta (Family 21) is heterozygous for a *DSPP* frameshift variant. A previous 2019 study found a novel frameshift variant in *DSPP* that caused dentin dysplasia II (14) which was similar to our study's patient's novel variant.

*PRKACB* codes for a Cβ-subunit which activates the protein kinase A (PKA) by binding to cyclic adenosine monophosphate (cAMP) molecules. PKA and cAMP create a pathway which plays an important role in endocrine system (hormonal and cellular) regulation. We recruited a patient (Family 22) presenting with oligodontia, ankylosis, and a congenital tooth absence. A novel *PRKACB* (c.1000A > C) was found, but of unknown pathogenicity. *PRKACB* variants have been found in association with dental anomalies before, notably hypodontia, conically-shaped teeth and congenital missing teeth (15).

Other previously reported variants were identified in *AMBN*, *PLOD1*, *EDAR*, *PRKAR1A*, *TSPEAR*, *FAM83H*, *DLX3*, *BMP2*, and *TGDS* genes (Online Supplementary Material).

Given our recruitment method (focused on a few geneticists and dentists so far), as well as our ongoing and sequential testing method (some only had limited panels, while others had exome sequencing), our study is not yet representative of the frequency of rare dental anomalies in the general population nor of the yield of specific tests per condition. Nevertheless, as we expand and continue our study, with more patients recruited, the Quebec Dental Anomalies Registry will be able to expand understanding of dental anomalies and their underlying genetic causes or syndromes. The clinical manifestations of these oral lesions in patients with rare diseases are often neglected, whereas they should be integrated into the universal health care for these disenabled patients with specific needs.

## Materials and methods

This study was approved by the Research Ethics Committee of the CHU Sainte-Justine (#DA Bank 1403). The inclusion criterion for the study was the presence of dental anomaly, defined as an anomaly of including teeth and surrounding structures such as the periodontium (alveolar bone, ligament, and gingivae). The data bank recruits patients suspected of having one or more dental anomalies through participating clinicians (e.g. dentists, doctors, orthodontists, etc.). Consenting (written) patients had specific genetic tests to determine if a variant associated with a genetic condition is present. For all families, we compile clinical data, family history, and either genetic test reports when diagnostic or DNA to conduct tests if no molecular cause is identified.

Genetic testing was done through single gene testing, genetic panel testing, or exome sequencing depending on the manifestations. Exome capture and high-throughput sequencing were performed at Génome Québec (Montréal, Canada). Using Agilent SureSelect all-exome kit (V4 optimized for Illumina HiSEQ sequencing), exomes were enriched with 2 µg of subjects’ genomic DNA. This enrichment was performed to cover about 50 Mb of genomic sequences, mainly protein coding sequences. Using Illumina HiSEQ 2000 platform (following the manufacturer's instructions), exon-enriched DNA libraries were sequenced (paired-end, 2 × 100 bp). Alignment and annotation of variants was performed by the DRAGEN Enrichment Pipeline (v3.9.5), then we used Illumina Variant Interpreter to filter for rare or new coding variants in genes already associated with diseases that may correspond to phenotypic elements of each patient, using hpo.jax.org and omim.org as a source of information for phenotypes associated with certain genes. Refer to Online Supplementary Table S2 for laboratories used for genetic testing. Cone beam imaging was performed as described in Ref. (16).

## Supplementary Material

pgad196_Supplementary_Data

## Data Availability

All data are included in the manuscript and/or Online Supplementary Material. Data and consents are compiled by Drs Moldovan and Campeau, and the Data Stewardship Committee is composed of these two investigators with the addition of Drs Schmittbuhl and Vu. All future data and samples compiled in the registry can be shared in an anonymized fashion with other investigators upon request, for the duration of our study, in accordance with the ethical standards of our institution. Reasonable requests for additional data and samples we have not compiled (such as additional imaging data or samples such as blood) can also be accommodated. Once published, the new genetic variants will be included in the ClinVar database.
