# Supplementary material for: The Quebec Dental Anomalies Registry: Identifying genes for rare disorders
Source: PNAS Nexus. 2023 Jun 14;2(6):pgad196. doi: 10.1093/pnasnexus/pgad196 (PMC10290489; doi:10.1093/pnasnexus/pgad196)
Supplement: pgad196_Supplementary_Data [file pgad196_supplementary_data.docx]

**
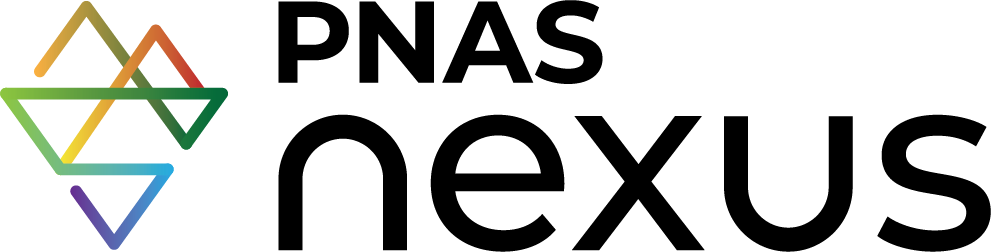
**

**Supplementary Information for**

The Quebec dental anomalies registry: identifying genes for rare disorders

Madeleine S Wredenhagen, Andee Goldstein, Hélène Mathieu, Valancy Miranda, Burcin Morali, Jacinthe Santerre, Catalina Maftei, Marie-Ange Delrue, Matthieu Schmittbuhl, Duy Dat Vu, Florina Moldovan, Philippe M Campeau

Corespondings autors : Philippe Campeau and Florina Moldovan

Email: [p.campeau@umontreal.ca](mailto:p.campeau@umontreal.ca) and [florina.moldovan@umontreal.ca](mailto:florina.moldovan@umontreal.ca)

**This PDF file includes:**

Supplementary text

Tables S1, S2 and S3

Legends for Datasets S1, S2 and S3

SI References

**Other supplementary materials for this manuscript include the following:**

**Supplementary Information Text**

Patient Family numbers are attributed based on their corresponding numbers in Table S1.

Many variants were found in different unrelated families, such as *WNT10A* c.803C>G presenting in three families (Families 1, 8 and 15), *WNT10A* c.682T>A presenting in three families (Families 1, 4 and 13, T1) and *WNT10A* c.321C>A presenting in two families (Families 7 and 8). Variants *WNT10A* c.682T>A and *WNT10A* c.321C>A have been previously identified in the literature. The c.682T>A variant was found in recruited patients with oligodontia as the only/main dental anomaly in varying degrees of severity, with or without non-dental anomalies. This variant, when found in a heterozygous state, was present in patients with a more severe diagnosis, even if the patient is heterozygous for the variant (Family 13). The c.682T>A variant with an additional *WNT10A* variant (compound heterozygous), either c.803C>G (Family 1) or c.1086C>A (Family 4), was associated with the least severe diagnosis of only oligodontia and a more severe diagnosis of Oligodontia Syndrome, respectively. A 2019 study by Ruiz-Heiland et al. (1) found that c.682T>A is present at a frequency of up to 2% in the general population and was the most frequent *WNT10A* variant in their patient group. In spite of being classified as pathogenic, diagnosis of patients with this variant range in severity (1) similarly to the patients observed in this study. It is therefore difficult to predict the phenotypic effect of this variant, especially when it is *in trans* with a second different variant.

The c.321C>A variant was found in recruited patients with a more severe clinical manifestations such as having multiple dental anomalies (in size, shape and number), and non-dental anomalies (thin hair, dysplastic nails, etc.) with either ectodermal dysplasia or Odonto-onycho-dermal Syndrome (a rare form of ectodermal dysplasia). Previous cases have been published where patients with Odonto-onycho-dermal Syndrome had a *WNT10A* c.321C>A variant (2). The severity of the symptoms depends on whether the patient is heterozygous or homozygous for the variant (2). In the Krøigård study (2), the patient presenting with ectodermal dysplasia is homozygous, whereas the patient in our cohort with Odonto-onycho-dermal Syndrome is compound heterozygous.

Variant *WNT10A* c.803C>G, has previous entries in ClinVar and was predicted as pathogenic by multiple *in* *silico* prediction tools (Mutation Taster, UMD-predictor, SIFT). The patients who are compound heterozygous for this variant as well as the c.682T>A variant present only with oligodontia as their dental anomaly and without non-dental anomalies (Family 1). Given that the c.682T>A variant is associated with a broad range of severity, it is difficult to identify the contribution of each variant to the patients’ diagnosis. However, the patient (Family 8) who is compound heterozygous for the c.803C>G and c.321C>A variants presents with multiple dental anomalies (including oligodontia) and non-dental anomalies. The Odonto-onycho-dermal Syndrome diagnosis of this patient is due to a combination of both variants. Given that the c.321C>A variant has been previously associated with Odonto-onycho-dermal Syndrome or ectodermal dysplasia, it appears more likely that this second variant is modifying the severity of the patient’s condition.

Four additional variants were found: *WNT10A* c.511C>T (Family 2), *WNT10A* c.1086C>A (Family 4), *WNT10A* c.1237G>A (Family 14) and *WNT10A* c.283G>A (Family 3). The *WNT10A* c.511C>T variant has been identified in the literature as more often associated with tooth agenesis such as anodontia and oligodontia (3). The variant is also prevalent in Chinese patients with allele frequency increasing in patients with more severe oligodontia [(3)](https://www.zotero.org/google-docs/?P6Twem). The patient recruited for our study is in accordance with the previous literature findings as his variant manifested mainly as oligodontia and a family history of anodontia. The c.283G>A variant has also been previously identified in literature and linked to isolated hypodontia (4), which is in accordance with our patient.

The *WNT10A* c.1086C>A and *WNT10A* c.1237G>A variants have not been previously identified in literature. The patients with c.1086C>A also have a c.682T>A variant which varies in severity. However, c.1086C>A was determined to be likely pathogenic, indicating that it probably contributed to the degree of severity of the patients’ condition. The patient with c.1237G>A is heterozygous for the variant and presents with multiple dental agenesis as the main dental anomaly with multiple non-dental anomalies. Because no other variant was identified in this patient, it is possible to interpret the condition of the patient as being caused solely by this new variant.

The *WNT10A* c.715C>T variant has not been previously identified in literature. The patient with this variant (Family 25) presents with a wide array of anomalies some of which we determined to be caused by the novel variant (oligodontia and hyperhidrosis) because of the previously established link in this study and literature between *WNT10A* and oligodontia. WNT10A is probably responsible for the hyperhydrosis because it is responsible for the formation of certain ectodermal structures during development, one of which are sweat glands (5). However more data, such as exome analysis, will be necessary in order to determine whether or not the other anomalies are being caused by this variant.

Amelogenesis imperfecta (type 1F), which is a group of genetic conditions that affect the enamel (6), is associated with variants of *AMBN* (7). Dental erosion is not a condition with a single cause,although variants in the genes responsible for enamel formation would increase the chances of being affected (8). The patient (Family 9) we recruited with both an *AMBN* (c.209C>G) and *PLOD1* (c.1097C>T) variant presented with severe dental erosion and a history of multiple fractures at a young age. The *AMBN* c.209C>G variant creates a premature stop codon which would impact the formation of the teeth’s enamel increasing the susceptibility of dental erosion. This variant is of unknown pathogenicity as this is a new variant not before described in literature but is a good candidate for the patient’s condition. *PLOD1* is associated with the kyphoscoliotic type of Ehlers-Danlos syndrome (EDS), a group of disorders caused by variants affecting collagen and therefore bones are cartilage. The patient’s history of multiple fractures at an early age may be a symptom of EDS. There is a type of EDS (pEDS) called the periodontal type that does affect the tissues surrounding and supporting the teeth but is associated with variants of two different genes (*C1R* and *C1S*). It is unclear if the patient’s *PLOD1* c.1097C>T could explain her phenotype as the pathogenicity of this variant is of unknown significance (VUS) according to ClinVar. It is also not clear whether *AMBN* and *PLOD1* contribute together to affect the patient’s dental phenotype, or both affect teeth and bones respectively.

The gene *EDAR* encodes the ectodysplasin A receptor, a receptor for the soluble ligand ectodysplasin A, a tumor necrosis factor. Variants in *EDAR* resulting in the malfunction of the gene impact the ectoderm’s tissues and structures leading to defects and forming a condition known as hypohidrotic ectodermal dysplasia (9). Ectodermal dysplasia is a group of genetic disorders affecting the tissues derived from the ectoderm (10). We recruited two patients of the same family (Family 6) with an *EDAR* variant, presenting with ectodermal dysplasia (with a family history), thin hair and anhidrosis. Both were found to be heterozygous for a c.1234C>T variant, which has not been described before in literature. This variant is a good candidate for the patients’ condition because of the known association of *EDAR* variants with ectodermal dysplasia.

Acrodysostosis is a rare peripheral dysostosis associated with accelerated bone age, developmental delays, brachydactyly, short stature and midface hypoplasia (11). Ankylosis is a condition in which the tooth’s cementum fuses to the surrounding alveolar bone. The periodontal ligament in that area is no longer present and is replaced by an osseous tissue. This condition renders that tooth immobile and therefore affects its eruption pattern. It is most common with primary molars; however, it can affect both primary and permanent dentition (12). There is a lot of uncertainty about the etiology of ankylosis. The patient recruited for this study (Family 12) has a *PRKAR1A* variant, presenting with ankylosis, superior incisor with root dysmorphisms, transposition tendency of germs and acrodysostosis as a non-dental anomaly. The 2013 study by Muhn et al. found novel variants of *PRKAR1A* associated with acrodysostosis, but not ankylosis, with only one of their patients presenting with a dental anomaly (oligodontia). The pathogenicity of *PRKAR1A* in regards to ankylosis remains unknown, and further studies are necessary to determine if there is a link between the ankylosis and acrodysostosis.

Ectodermal dysplasia and tooth agenesis have both been associated with variants in the *TSPEAR* gene (important for tooth and nail development). The severity of the affected individual’s phenotype varies widely, and although *TSPEAR* variants are commonly accompanied by hearing loss, this is not always the case when the gene causes ectodermal dysplasia (13). The patient recruited (Family 10), homozygous for a *TSPEAR* c.974delC variant, was diagnosed with ectodermal dysplasia secondary to *TSPEAR* variant. The patient’s symptoms align with previous literature on ectodermal dysplasia related to *TSPEAR* variants. This variant has not been described in the literature before but is interpreted as likely pathogenic if not pathogenic given that it leads to a frameshift and was determined to be the cause of the patient’s condition.

Variants of the *FAM83H* gene are involved with amelogenesis imperfecta (AI), specifically autosomal-dominant hypocalcified AI, which is a severe form of the disease. *FAM83H* codes for extracellular matrix proteins and is the first gene of that type to be associated with AI (Family 19) (14).

*DLX3* codes for a protein expressed in ectodermal tissues and plays an important part in their development. *DLX3* regulated *DSPP* expression, and together this controls dentin formation. Variants in *DLX3* have shown to result in severe enamel and dentin defects because of their inability to act as transcription factors for *DSPP* (15). The patient recruited for this study (Family 20) has AI of all primary teeth (a condition that affects the enamel) and a *DLX3* (c.376C>T) variant. This novel variant does correspond to existing studies linking *DLX3* to enamel/dentin defects.

The *BMP2* gene variant found in our patient (Family 23) has been previously reported in literature. The phenotypes associated with this gene and variant correlate with our patient’s phenotype, such as the “Short stature, facial dysmorphism and skeletal anomalies with or without cardiac anomalies 1” syndrome and possible brachydactyly (16).

Both variants in *TGDS* have been previously reported in literature. The *TGDS* gene and its subsequent variants have previously been associated with the Pierre-Robin sequence or more specifically: Cantel-Manzke syndrome (both in typical and atypical patients). Our patient (Family 24) presented with a very similar phenotype as the ones described in previous articles regarding *TGDS* variants (17).

**Table S1.** Clinical Summary

| F. | Ind. | Dental anomalies | Non-dental anomalies | Results |
| --- | --- | --- | --- | --- |
| 1 | 1 | Oligodontia (with family history) | None | Compound htz NM_025216.3 *WNT10A* variants pathogenic c.803C>G; p.Ser268Ter and likely pathogenic c.682T>A; p.Phe228Ile  Odonto-onycho-dermal dysplasia |
|  | 2 | Oligodontia (with family history) | None | Compound htz NM_025216.3 *WNT10A* variants pathogenic c.803C>G; p.Ser268Ter and likely pathogenic c.682T>A; p.Phe228Ile  Odonto-onycho-dermal dysplasia |
|  | 3 | Oligodontia (with family history) | None | Compound htz NM_025216.3 *WNT10A* variants pathogenic c.803C>G; p.Ser268Ter and likely pathogenic c.682T>A; p.Phe228Ile  Odonto-onycho-dermal dysplasia |
| 2 | 1 | Isolated oligodontia of seven teeth  Family history of oligodontia | Thin hair | Htz likely pathogenic (dominant with a limited penetrance) NM_025216.3 *WNT10A* variant c.511C>T; p.Arg171Cys |
|  | 2 | Oligodontia | None | Carrier for *WNT10A* variant c.511C>T; p.Arg171Cys |
| 3 | 1 | Isolated oligodontia, congenital absence of eight teeth, primary teeth retention, dental malocclusion, shape anomalies, and a moderated lack of alveolar growth. | None | Htz likely pathogenic (dominant autosomal) NM_025216.3 *WNT10A* variant c.283G>A; Glu95Lys |
| 4 | 1 | Oligodontia of 11 to 15 permanent teeth. | Global delayed development, asthma, pneumonias, hypospadias, dysplastic nails, hypersudation of the feet, pectus excavatum, and spatulated fingers | Compound htz likely pathogenic NM_025216.3 *WNT10A* variants c.682T>A; p.Phe228Ile and c.1086C>A; p.Cys362Ter  Diagnosis of Oligodontia Syndrome |
|  | 2 | Oligodontia of 6 to 8 permanent teeth | Hyperlaxity in the small articulations | Compound htz likely pathogenic NM_025216.3 *WNT10A* variants c.682T>A; p.Phe228Ile and c.1086C>A; p.Cys362Ter |
| 5 | 1 | Microdontia, oligodontia (only eight adult teeth left), with a family history. | Wiry hair, but with no evidence of affect skin | Htz VUS micro-duplication of at least 6.6 kb including the entire *KRT85* gene  Condition remains unsolved |
| 6 | 1 | Ectodermal dysplasia with a family history | Thin hair and anhidrosis | Htz likely pathogenic NM_022336.4 *EDAR* variant c.1234C>T; p.Leu412Phe |
|  | 2 | Ectodermal dysplasia with a family history | Thin wiry hair and anhidrosis | Htz likely pathogenic NM_022336.4 *EDAR* variant c.1234C>T; p.Leu412Phe |
| 7 | 1 | Ectodermal dysplasia, conical spaced teeth, delayed eruption of certain teeth, but with all teeth present | Thin hair, and fragile dysplastic nails on both hands and feet | Hmz pathogenic NM_025216.3 *WNT10A* variant c.321C>A; p.Cys107Ter |
| 8 | 1 | Odonto-onycho-dermal Syndrome; multiple dental anomalies, oligodontia, and deformed pointy primary teeth. | Hyperlaxity, clinodactyly of the fifth finger, hypersudation of the hands and feet in adolescence, dry and brittle hair, flat fragile and striated nails | Compound htz NM_025216.3 *WNT10A* variants pathogenic c.321C>A; p.Cys107Ter and likely pathogenic c.803C>G; p.Ser268Ter |
| 9 | 1 | Severe dental erosion | History of multiple fractures at a young age | Htz pathogenic NM_016159.6 *AMBN* variant c.209C>G; p.Ser70Ter  Htz VUS NM_000302.4 *PLOD1* variant c.1097C>T; p.Ala366Val |
| 10 | 1 | Ectodermal dysplasia, microdontia, supernumerary lower tooth, severe oligodontia of 12 missing adult teeth: #12-13, #17, #22-23, #27, #31-32, #37, #41-42, and #47. Cone-like shapes of the canines and premolars. Root dysmorphism with disto-lingual roots on each molar (#16 and #26), and dental rhizalysis of teeth #65 and #75 | Alacrima at a young age, late hair growth (five years of age) and thin nails. | Hmz pathogenic NM_144991.3 *TSPEAR* variant c. 974delC; p.Ser325Ter  Both parents are carriers |
| 11 | 1 | Dentinal dysplasia, incorrect orientation of the permanent dentition, supernumerary teeth around incisors with “ghost” roots. No primary dentition defects other than a few cavities | Thrombosis of the Galen vein, delayed fine motor skills, but no signs of intellectual disability | Type 1 dentinal dysplasia. Negative DSPP sequencing; test did not include sequencing within the large repeat element in exon 5. Additional analyses need to be done. |
| 12 | 1 | Ankylosis, superior incisor with root dysmorphisms (short root #11-21). and transposition tendency of germs #15-25 in relation to germs #14-24. | Acrodysostosis | Htz likely pathogenic NM_212472.2 *PRKAR1A* variant c.379_380delinsTT; p.Ala127Phe. |
| 13 | 1 | Oligodontia and an isolated diagnostic impression of multiple agenesis of type 4 in ten adult teeth (third molars, molars, and premolars) | ADHD, eczema, anxiety and clinodactyly of the third bilateral finger | Htz likely pathogenic NM_025216.3 *WNT10A* variant c.682T>A; p.Phe228Ile |
| 14 | 1 | Multiple dental agenesis, congenital absence of six teeth (four premolars on the upper jaw and two on the lower jaw) and retention of primary teeth | Fructose intolerance, dermographism, Hallux valgus and fragile nails | Htz pathogenic NM_025216.3 *WNT10A* variant c.1237G>A; p.Val413Ile |
| 15 | 1 | Oligodontia of 2 primary and 11 permanent teeth | None | Htz Pathogenic NM_025216.3 *WNT10A* variant c. 803C>G; p. Ser268Ter |
| 16 | 1 | Oligodontia of 9 teeth | None | No candidate variant identified |
| 17 | 1 | Ankylosed tooth (#85) and a congenital missing #45 tooth. | None | No candidate variant identified |
| 18 | 1 | Peg shaped tooth (#22) and three congenital missing teeth. | None | Genetic analyses not performed yet |
| 19 | 1 | Amelogenesis imperfecta (seemingly isolated) | None | Htz VUS NM_198488.5 *FAM83H* variant c.1436G>A; p.Gly479Asp  Htz 31.3kb deletion in the *ITGB6* gene |
| 20 | 1 | Amelogenesis imperfecta of all primary teeth | None | Htz likely pathogenic NM_005220.3 *DLX3* variant c.376C>T; p.Pro126Ser |
| 21 | 1 | Dentinogenesis imperfecta | None | Htz likely pathogenic NM_014208.3 *DSPP* variant c.1439_1454del; p.Glu480AlafsTer829 |
| 22 | 1 | Oligodontia, ankylosed tooth (#85) and congenital absence of tooth #45 | N/A | Htz likely pathogenic NM_182948.4 *PRKACB* variant c.1000A>C; p.Asn334His |
| 23 | 1 | Narrow jaw, late apparition of adult teeth and a tooth alignment problem | Scoliosis, hallux valgus, large cranial parameter, narrow palate, bifid uvula, inverted nipples, and short and wide hands with possible brachydactyly | Htz pathogenic NM_001200.4 *BMP2* variant c.142G>T; p.Glu48Ter  “Short stature, facial dysmorphism and skeletal anomalies with or without cardiac anomalies 1” syndrome |
| 24 | 1 | Malocclusion, dental extraction, misalignment of inferior teeth | Arterial hypertension, bilateral clinodactyly, severe Pierre-Marie-Robin sequence with a cleft palate, difficulties with feeding, gavage at a young age, vesicoureteral reflux at a young age, staturo-ponderal growth delay, slight developmental delay, and a large cardiac ventricular septal defect with a slight pulmonary stenosis | Compound htz likely pathogenic NM_014305.4 *TGDS* variants likely pathogenic c.298G>T; p.Ala100Ser (paternal) and likely pathogenic c.269A>G; p.Glu90Gly (maternal)  Cantel-Manzke syndrome |
| 25 | 1 | Oligodontia (missing five teeth and wisdom teeth), retrognathia | Narrow and high-arched palate, slight gingival hypertrophy, predominant thenar hypotrophy of the left side, slight muscular hypotrophy of the left forearm, small bilaterally fingers with some having a pronounced hyperlaxity of the interphalangeal joints, limitations in the flexibility of the wrists (predominantly the left one), short and stocky feet with a metatarsus varus positional tendency, hyperhidrosis of the hands and feet with a slight plantar hyperkeratosis, distal arthrogryposis of the upper limbs and glasses at a young age to correct astigmatism | Htz VUS NM_025216.3 *WNT10A* variant c.715C>T; p.His239Tyr (maternal) |

The following abbreviations are used: F., Family; Ind., Individual; Htz, Heterozygous; Hmz, Homozygous; VUS, Variant of unknown significance; N/A, not available.

**Table S2**. Laboratories and genes tested

| Family | Individual | Laboratory for genetic testing | Gene(s) tested |
| --- | --- | --- | --- |
| 1 | 1 | GeneDx | *WNT10A* (targeted test for familial variants) |
|  | 2 | GeneDx | *WNT10A* (targeted test for familial variants) |
|  | 3 | GeneDx | *WNT10A* (targeted test for familial variants) |
| 2 | 1 | CTGT | *AXIN2, EDA, LRP6, LTBP3, MSX1, PAX9, PTH1R, WNT10A, WNT10B (*NGS + del/dup) |
|  | 2 | CTGT | *WNT10A* (targeted test for familial variants) |
| 3 | 1 | CTGT | *AXIN2, EDA, LRP6, LTBP3, MSX1, PAX9, PTH1R, WNT10A, WNT10B (*NGS *+* del/dup*)* |
| 4 | 1 | PREVENTION | *AXIN2, EDA, EDAR, EDARADD, LTBP3, MSX1, PAX9, WNT10A (*NGS + del/dup*)* |
|  | 2 | PREVENTION | *WNT10A* (targeted test for familial variants) |
| 5 | 1 | CTGT | *EDA, EDAR, EDARADD, GJB6, HOXC13, KDF1, KREMEN1, KRT74, KRT85, MSX1 (*NGS + del/dup*)* |
| 6 | 1 | PREVENTION | *EDA, EDAR, EDARADD, KRT85, NECTIN1, WNT10A (*NGS + del/dup*)* |
|  | 2 | PREVENTION | *EDAR* (targeted test for familial variants) |
| 7 | 1 | BLUEPRINT | *BCS1L, CDH3, DSP, EDA, EDAR, EDARADD, ERCC2, EVC, EVC2, GJB2, GJB6, HOXC13, HR, IFT122, JUP, LRP6, MPLKIP, PORCN, PRKD1, RMRP, TP63, WDR35, WNT10A (*NGS + del/dup*)* |
| 8 | 1 | PREVENTION | *Prevention, AXIN2, EDA, EDAR, EDARADD, LTBP3, MSX1, PAX9, WNT10A (*NGS + del/dup*)* |
| 9 | 1 | PREVENTION | *ACP4, ALPL, AMBN, AMER1, AMTN, ASCC1, B3GAT3, B4GALT7, BMP1, CA2, CDK5RAP2, CLCN5, CLCN7, COL1A1, COL1A2, CREB3L1, CRTAP, CYP27B1, CYP2R1, DMP1, DSPP, ENPP1, FERMT3, FGF23, FKBP10, GPAA1, IFITM5, LRP5, MBTPS2, MEPE, MESD, NBAS, NTRK1, OSTM1, P3H1, PHEX, PLOD2, PLS3, PPIB, PRKAR1A, RELT, SEC24D, SERPINF1, SERPINH1, SGMS2, SH3PXD2B, SLC29A3, SLC2A2, SLC34A1, SLC34A3, SLC9A3R1, SNX10, SOST, SP7, SPARC, TAPT1, TCIRG1, TENT5A, TGFB1, TMEM38B, TNFRSF11A, TNFRSF11B, TNFSF11, TRIP4, WNT1, XYLT2* with raw data revision |
| 10 | 1 | PREVENTION | *EDA, EDAR, EDARADD, KRT85, NECTIN1, WNT10A* (NGS + del/dup) with raw data revision |
| 11 | 1 | PREVENTION | *DSPP* |
| 12 | 1 | PREVENTION // Fulgent | *TRPS1* (seq + aCGH) // *ADAMTSL2, FBN1, PDE4D, PRKAR1A, RMRP, SMAD4* (NGS) |
| 13 | 1 | PREVENTION | *AXIN2, EDA, EDAR, EDARADD, LTBP3, MSX1, PAX9, WNT10A* (NGS + del/dup) |
| 14 | 1 | PREVENTION | *AXIN2, EDA, EDAR, EDARADD, KREMEN1, LRP6, LTBP3, MMP20, MSX1, PAX9, WNT10A, WNT10B* (NGS + del/dup) |
| 15 | 1 | PREVENTION | *AXIN2, EDA, EDAR, EDARADD, LTBP3, MSX1, PAX9, WNT10A* |
| 16 | 1 | N/A | Exome |
| 17 | 1 | N/A | Exome |
| 18 | 1 | N/A | N/A |
| 19 | 1 | PREVENTION | *AMELX, CNNM4, DLX3, ENAM, FAM20A, FAM83H, GPR68, ITGB6, KLK4, LAMA3, LAMB3, LTBP3, MMP20, ODAPH, ROGDI, SLC24A4, SMOC2, WDR72* |
| 20 | 1 | N/A | Exome |
| 21 | 1 | N/A | Exome |
| 22 | 1 | N/A | Exome |
| 23 | 1 | PREVENTION | *FBN1, SKI, SMAD2, SMAD3, TGFB2, TGFB3, TGFBR1, TGFBR2, TP63* |
| 24 | 1 | N/A | Exome |
| 25 | 1 | PREVENTION | *AXIN2, CHST14, ECEL1, EDA, EDAR, EDARADD, FBN2, LTBP3, MSX1, MYBPC1, MYH3, MYH8, NALCN, PAX9, PIEZO2, TNNI2, TNNT3, TPM2, WNT10A* |

Patient Family numbers are attributed based on their corresponding numbers in Table S1

The following abbreviations are used: N/A, not available.

**Table S3.** Variant Summary

| Patients | NM_ | Mutations | Amino acid change | Variants in published articles | ACMG classification | Previously reported classification | Our interpretation |
| --- | --- | --- | --- | --- | --- | --- | --- |
| Family 1 | 025216.3 | *WNT10A* c.803C>G | p.Ser268Ter | [(Tziotzios et al. 2014)](https://www.zotero.org/google-docs/?OYC2j2) (19) | Pathogenic | Pathogenic | Pathogenic |
|  |  | *WNT10A* c.682T>A | p.Phe228Ile | [(Bohring et al. 2009)](https://www.zotero.org/google-docs/?PAiC3L) (18) | VUS | CIP  Likely benign  Pathogenic | Likely pathogenic |
| Family 2 | 025216.3 | *WNT10A* c.511C>T | p.Arg171Cys | [(He et al. 2013)](https://www.zotero.org/google-docs/?uUG0P7) (20) | VUS | CIP  Benign  Likely benign  Likely pathogenic | Likely pathogenic Dominant with reduced penetrance |
| Family 3 | 025216.3 | WNT10A c.283G>A | p.Glu95Lys | [(Boogaard et al. 2012)](https://www.zotero.org/google-docs/?pQlD2U) (4) | Pathogenic | VUS  Likely pathogenic  Pathogenic | Likely pathogenic |
| Family 4 | 025216.3 | *WNT10A* c.682T>A | p.Phe228Ile | [(Bohring et al. 2009)](https://www.zotero.org/google-docs/?CS43bM) (18) | VUS | CIP  Likely benign  Pathogenic | Likely pathogenic |
|  | 025216.3 | *WNT10A* c.1086C>A | p.Cys362Ter | N/A | Likely pathogenic | N/A | Likely pathogenic |
| Family 6 | 022336.4 | *EDAR* c. 1234C>T | p.Leu412Phe | N/A | VUS | N/A | Likely pathogenic |
| Family 7 | 025216.3 | *WNT10A* c.321C>A | p.Cys107Ter | [(Bohring et al. 2009)](https://www.zotero.org/google-docs/?6wFBpw) (18) | Pathogenic | VUS  Pathogenic | Pathogenic |
| Family 8 | 025216.3 | *WNT10A* c.321C>A | p.Cys107Ter | [(Bohring et al. 2009)](https://www.zotero.org/google-docs/?jwiZtj) (18) | Pathogenic | VUS  Pathogenic | Pathogenic |
|  | 025216.3 | *WNT10A* c.803C>G | p.Ser268Ter | [(Tziotzios et al. 2014)](https://www.zotero.org/google-docs/?XNUFST) (19) | Pathogenic | Pathogenic | Likely Pathogenic |
| Family 9 | 016519.6 | *AMBN* c.209C>G | p.Ser70Ter | N/A | VUS | N/A | Pathogenic |
|  | 000302.4 | *PLOD1* c.1097C>T | p.Ala366Val | [(Nykamp et al. 2017)](https://www.zotero.org/google-docs/?CYRoVb) (21) | VUS | VUS | VUS |
| Family 10 | 144991.3 | *TSPEAR* c.974delC | p.Ser325Ter | N/A | Pathogenic | N/A | Pathogenic |
| Family 12 | 212472.2 | *PRKAR1A* c.379_380delinsTT | p.Ala127Phe | N/A | VUS | N/A | Likely pathogenic |
| Family 13 | 025216.3 | *WNT10A* c.682T>A | p.Phe228Ile | [(Bohring et al. 2009)](https://www.zotero.org/google-docs/?broken=53XUno) (18) | VUS | CIP  Likely benign  Pathogenic | Likely pathogenic |
| Family 14 | 025216.3 | *WNT10A* c.1237G>A | p.Val413Ile | N/A | VUS | N/A | Pathogenic |
| Family 15 | 025216.3 | *WNT10A* c.803C>G | p.Ser268Ter | [(Tziotzios et al. 2014)](https://www.zotero.org/google-docs/?vavMPr) (19) | Pathogenic | Pathogenic | Pathogenic |
| Family 19 | 198488.5 | *FAM83H* c.1436G>A | p.Gly479Asp | N/A | VUS | N/A | VUS |
| Family 20 | 005220.3 | *DLX3* c.376C>T | p.Pro126Ser | N/A | VUS | N/A | Likely pathogenic |
| Family 21 | 014208.3 | *DSPP* c.1439_1454del | p.Glu480AlafsTer829 | N/A | Likely pathogenic | N/A | Likely pathogenic |
| Family 22 | 182948.4 | *PRKACB* c.1000A>C | p.Asn334His | N/A | Likely Pathogenic | N/A | Likely pathogenic |
| Family 23 | 001200.4 | *BMP2* c.142G>T | p.Glu48Ter | (Tan et al. 2017) (16) | Pathogenic | Pathogenic | Pathogenic |
| Family 24 | 014305.4 | *TGDS* c.298G>T | p.Ala100Ser | (Pferdehirt et al 2015) (22) | Likely Pathogenic | Pathogenic  Likely Pathogenic | Likely Pathogenic |
|  | 014305.4 | *TGDS* c.269A>G | p.Glu90Gly | (Ehmke et al. 2014) (23) | VUS | Pathogenic | Likely Pathogenic |
| Family 25 | 025216.3 | *WNT10A* c.715C>T | p.His239Tyr | N/A | VUS | N/A | VUS |

Patient Family numbers are attributed based on their corresponding numbers in Table S1

The following abbreviations are used: CIP, Conflicting interpretations of pathogenicity; VUS, Variant of unknown significance; N/A, not available.

**SI References**

1. Ruiz-Heiland G, Lenz S, Bock N, Ruf S. Prevalence of WNT10A gene mutations in non-syndromic oligodontia. *Clin Oral Investig*. 2019;23(7):3103-3113. doi:10.1007/s00784-018-2731-4
2. Krøigård AB, Clemmensen O, Gjørup H, Hertz JM, Bygum A. Odonto-onycho-dermal dysplasia in a patient homozygous for a WNT10A nonsense mutation and mild manifestations of ectodermal dysplasia in carriers of the mutation. *BMC Dermatol*. 2016;16:3. doi:10.1186/s12895-016-0040-7
3. Song S, Zhao R, He H, Zhang J, Feng H, Lin L. WNT10A variants are associated with non-syndromic tooth agenesis in the general population. *Hum Genet*. 2014;133(1):117-124. doi:10.1007/s00439-013-1360-x
4. van den Boogaard MJ, Créton M, Bronkhorst Y, et al. Mutations in WNT10A are present in more than half of isolated hypodontia cases. *J Med Genet*. 2012;49(5):327-331. doi:10.1136/jmedgenet-2012-100750
5. Doolan BJ, Onoufriadis A, Kantaputra P, McGrath JA. WNT10A, dermatology and dentistry. *Br J Dermatol*. 2021;185(6):1105-1111. doi:10.1111/bjd.20601
6. Crawford PJ, Aldred M, Bloch-Zupan A. Amelogenesis imperfecta. *Orphanet J Rare Dis*. 2007;2:17. Published 2007 Apr 4. doi:10.1186/1750-1172-2-17
7. Lamandé SR, Bateman JF. Genetic Disorders of the Extracellular Matrix. *Anat Rec (Hoboken)*. 2020;303(6):1527-1542. doi:10.1002/ar.24086
8. Søvik JB, Vieira AR, Tveit AB, Mulic A. Enamel formation genes associated with dental erosive wear. *Caries Res*. 2015;49(3):236-242. doi:10.1159/000369565
9. Park JH, Yamaguchi T, Watanabe C, et al. Effects of an Asian-specific nonsynonymous EDAR variant on multiple dental traits. *J Hum Genet*. 2012;57(8):508-514. doi:10.1038/jhg.2012.60
10. Meshram GG, Kaur N, Hura KS. A case report of hypohidrotic ectodermal dysplasia: A mini-review with latest updates. *J Family Med Prim Care*. 2018;7(1):264-266. doi:10.4103/jfmpc.jfmpc_20_17
11. Muhn F, Klopocki E, Graul-Neumann L, et al. Novel mutations of the PRKAR1A gene in patients with acrodysostosis. *Clin Genet*. 2013;84(6):531-538. doi:10.1111/cge.12106
12. Mortazavi H, Baharvand M. Review of common conditions associated with periodontal ligament widening. *Imaging Sci Dent*. 2016;46(4):229-237. doi:10.5624/isd.2016.46.4.229
13. Bowles B, Ferrer A, Nishimura CJ, et al. TSPEAR variants are primarily associated with ectodermal dysplasia and tooth agenesis but not hearing loss: A novel cohort study. *Am J Med Genet A*. 2021;185(8):2417-2433. doi:10.1002/ajmg.a.62347
14. Xin W, Wenjun W, Man Q, Yuming Z. Novel FAM83H mutations in patients with amelogenesis imperfecta. *Sci Rep*. 2017;7(1):6075.doi:10.1038/s41598-017-05208-0
15. Duverger O, Zah A, Isaac J, et al. Neural crest deletion of Dlx3 leads to major dentin defects through down-regulation of Dspp. *J Biol Chem*. 2012;287(15):12230-12240. doi:10.1074/jbc.M111.326900
16. Tan TY, Gonzaga-Jauregui C, Bhoj EJ, et al. Monoallelic BMP2 Variants Predicted to Result in Haploinsufficiency Cause Craniofacial, Skeletal, and Cardiac Features Overlapping Those of 20p12 Deletions. *Am J Hum Genet*. 2017;101(6):985-994. doi:10.1016/j.ajhg.2017.10.006
17. Boschann F, Stuurman KE, de Bruin C, et al. TGDS pathogenic variants cause Catel-Manzke syndrome without hyperphalangy. *Am J Med Genet A*. 2020;182(3):431-436. doi:10.1002/ajmg.a.61419
18. Bohring A, Stamm T, Spaich C, et al. WNT10A mutations are a frequent cause of a broad spectrum of ectodermal dysplasias with sex-biased manifestation pattern in heterozygotes. *Am J Hum Genet*. 2009;85(1):97-105. doi:10.1016/j.ajhg.2009.06.001
19. Tziotzios C, Petrof G, Liu L, et al. Clinical features and WNT10A mutations in seven unrelated cases of Schöpf-Schulz-Passarge syndrome. *Br J Dermatol*. 2014;171(5):1211-1214. doi:10.1111/bjd.13158
20. He H, Han D, Feng H, et al. Involvement of and interaction between WNT10A and EDA mutations in tooth agenesis cases in the Chinese population. *PLoS One*. 2013;8(11):e80393. doi:10.1371/journal.pone.0080393
21. Nykamp K, Anderson M, Powers M, et al. Sherloc: a comprehensive refinement of the ACMG-AMP variant classification criteria [published correction appears in Genet Med. 2020 Jan;22(1):240-242]. *Genet Med*. 2017;19(10):1105-1117. doi:10.1038/gim.2017.3
22. Pferdehirt R, Jain M, Blazo MA, Lee B, Burrage LC. Catel-Manzke Syndrome: Further Delineation of the Phenotype Associated with Pathogenic Variants in *TGDS*. *Mol Genet Metab Rep*. 2015;4:89-91. doi:10.1016/j.ymgmr.2015.08.003
23. Ehmke N, Caliebe A, Koenig R, et al. Homozygous and compound-heterozygous mutations in TGDS cause Catel-Manzke syndrome. *Am J Hum Genet*. 2014;95(6):763-770. doi:10.1016/j.ajhg.2014.11.004
